# Improving the Meat and Meat Product Quality of Rare Pork Breeds and Genetic Types

**DOI:** 10.3390/foods13121901

**Published:** 2024-06-17

**Authors:** Alfredo Teixeira, Javier Álvarez-Rodríguez

**Affiliations:** 1 Centro de Investigação de Montanha (CIMO), Instituto Politécnico de Bragança, Campus de Santa Apolónia, 5300-253 Bragança, Portugal; 2 Laboratório para a Sustentabilidade e Tecnologia em Regiões de Montanha, Instituto Politécnico de Bragança, Campus de Santa Apolónia, 5300-253 Bragança, Portugal; 3 Department of Animal Science, University of Lleida, 25198 Lleida, Spain; javier.alvarez@udl.cat

Pork is the most consumed meat globally, particularly in Asia, Europe, and America [1]. However, production has been focused on lean genotypes. Breeds and genetic types with different body lipid deposition patterns have been under-researched regarding their carcass grading and the physical, chemical, and sensory qualities of their meat and processed products, such as cooked, dry-cured, and smoked varieties. On the other hand, the commercial value of the carcasses, the organoleptic, nutritional, and technological properties of the meat (i.e., suitability for processing and storage), convenience, and the social image in its cultural, ethical, and environmental dimensions related to pork production, including geographical origin, all influence society’s perceptions of pork [2]. These products are significant in traditional and ethnic cuisines and play an integral role in the gastronomic cultures of many regions [3]. The ranking order for pork production preferences was quality and health, respect for the environment and animals, regional identity, and production efficiency [4]. Recently, there has been growing interest in these genotypes to produce niche pork products with added credence attributes and economic value [3].

Also, according to Stange et al. [5], the study of interactions between genetic loci within and between species will play a fundamental role in understanding the potential for adaptations of species and communities and their effects on biodiversity, ecosystems, and people. This trend aligns with the United Nations’ sustainable development goals, emphasizing the importance of genetic diversity in food production and responding to current consumer demands [6]. Improving the quality of meat and meat products from rare pork breeds and genetic types is a multifaceted approach, including genetic selection, crossbreeding, and advanced techniques of animal nutrition and meat processing. In this Special Issue, researchers submitted original studies exploring new strategies to enhance the quality of meat and meat products from non-standard pork breeds. Seventeen manuscripts were submitted, and twelve were published. These studies, conducted by research groups worldwide with diverse scientific backgrounds, provide valuable insights into several research fields on rare pig breeds and genetic types.

The different studies can be categorized into four research subjects:

1. Carcass and meat quality;
2. Nutrition and effects of different ingredient supplements;
3. Genetics.

*Carcass and meat quality.*

Regarding meat and meat products, Leite et al. (contributions 1 and 2) studied the quality of three products (loin, “cachaço”, and shoulder) and the meat and fat quality (contribution 3) of Bísaro pork, a Portuguese breed, as well as some healthy meat aspects such as the reduction in salt and the absence of nitrite in the cure process. Beyond the physicochemical characterization, the studies also provide information about the sensory quality of the products. The meat quality of Nero d’Abruzzo pig, a native breed of Central Italy, in comparison with commercial meat products obtained from hybrid pigs was given in a study conducted by Ianni et al. and Roberts et al. (contributions 4 and 5, respectively). Their studies on the growth performance, body composition, and meat quality of Mangalica pigs suggest that higher price points for Mangalica pork in niche markets are justified. Their research demonstrates the unique qualities of Mangalica pork, which command premium prices due to superior marbling and tenderness compared to conventional pork. Also, in relation to the Mangalica breed, Charlton et al. (contribution 6) studied the optimal harvest weight, which is between 82 and 102 kg. In a study examining the meat processing characteristics between Duroc-sired (DS) and heritage breed Large Black (LB) pigs, contribution 8 pointed out that methods to improve lean percentage and processing yields while preserving potential desirable flavor traits in LB pigs would be beneficial to improve product quality and probable commercial integration.

*Nutrition and effects of different ingredient supplements.*

Working with Bísaro, the effect of different olive cakes on the pig diet was studied from the perspective of using highly polluting waste from the olive oil extraction industry (contributions 1 and 3). The effects of dietary betaine supplementation on growth performance, meat quality, and the muscle lipid metabolism of growing–finishing pigs were studied by Fu et al. (contribution 8), and they concluded that betaine supplementation at 1200 mg/kg could increase the growth performance of growing–finishing pigs and improve the meat quality and IMF content. Also, the effects of perilla cake supplementation in a low-lysine diet on Thai crossbred finishing pigs’ productivity, carcass and meat quality, and fatty acid composition were evaluated by Sringarm et al. (contributions 9); they verified that the meat quality was improved, particularly intramuscular fat, as well as the marbling score. However, the mechanism by which perilla cake combined with a low lysine content alters fatty acid deposition in pigs requires further investigation.

*Genetics*.

One study aimed to identify the related candidate genes of backfat quality and to preliminary clarify the molecular regulatory mechanism underlying pig backfat quality phenotypes; it was performed in Beijing Black pigs by Liu et al. (contribution 10), enhancing the current understanding of these mechanisms and providing a foundation for further studies. Predicting that the skeletal muscle fiber types can affect pork quality parameters, Wei et al. (contribution 11) used an isobaric Tag for Relative and Absolute Quantification (iTRAQ)-based proteome in three full-sibling Duroc × Meishan female pigs to identify the key proteins affecting the skeletal muscle fiber types. The authors found 12 genes overlapping between differentially expressed genes and differentially abundant proteins, which are the key candidate genes regulating the formation of skeletal muscle fibers. Pei et al. (contribution 12) determined the lipidomics of subcutaneous fat in myostatin (MSTN) single-copy mutant pigs and evaluated the variations in the lipid contents of the subcutaneous fat from MSTN+/− and wild-type Large White pigs via ultra-performance liquid chromatography–quadrupole/Orbitrap mass spectrometry. The results confirmed that MSTN participates in the regulation of fat metabolism, and the reduced expression of MSTN can ultimately influence the accumulation of lipid contents in the subcutaneous fat of pigs.
